# Factors Associated With Not Receiving Mental Health Services Among Children With A Mental Disorder in Early Childhood in the United States, 2021–2022

**DOI:** 10.5888/pcd21.240126

**Published:** 2024-10-10

**Authors:** Julie Fang Meng, Eileen Wiznitzer

**Affiliations:** 1 Belmont High School, Belmont, Massachusetts

## Abstract

**Introduction:**

Many mental disorders begin in early childhood. Without timely treatment, mental disorders experienced by young children can impair their learning ability and relationships with others, causing lifelong complications. However, not all children with a mental disorder in early childhood receive treatment.

**Methods:**

Using data collected from 46,424 children aged 2 to 8 years in the 2 most recent cycles of the National Survey of Children’s Health (2021 and 2022), we estimated the prevalence of having a mental disorder and investigated factors associated with young children not receiving mental health care when needed. All analyses were adjusted for survey weights to account for the complex sampling design and nonresponse biases in generating nationally representative estimates.

**Results:**

In 2021 and 2022, 19.0% of US children aged 2 to 8 years had 1 or more mental disorders. Of these children, 9.1% reported not receiving any needed health care in the previous 12 months, and of these, 45.8% reported not receiving mental health services when needed. The primary reasons for not receiving needed health care were problems getting an appointment (72.1%), issues related to cost (39.3%), and services needed not being available in the area (38.5%). Poor experiences with health care providers were consistently associated with not receiving needed mental health services among children with mental disorders.

**Conclusion:**

Our findings suggest a strong link between health care factors and not receiving needed mental health services among US children with a mental disorder in early childhood. In addition to increasing the availability of mental health services and expanding health insurance coverage, future public health efforts should prioritize enhancing patients’ experiences with health care providers.

**Summary**

**What is already known on this topic?**

Many mental disorders begin in early childhood. Without timely treatment, mental disorders experienced by young children can impair their learning ability and relationships with others, causing lifelong complications.

**What is added by this report?**

Using data collected in 2021 and 2022 from a large, nationally representative sample of US children, we estimated that 19.0% of children aged 2 to 8 years had 1 or more mental disorders. Poor experiences with health care providers were consistently associated with not receiving mental health services when needed among young children with mental disorders.

**What are the implications for public health practice?**

Future public health efforts should prioritize enhancing patients’ experiences with mental health care providers.

## Introduction

One in 5 children aged 3 to 17 years in the US has a mental disorder (1). Mental disorders in children are characterized by a clinically significant disturbance in a child’s cognition, emotion, or behavior and often include mental, behavioral, and developmental disorders (2). According to national surveys, the most common mental disorders affecting children in the US are anxiety, depression, attention-deficit/hyperactivity disorder (ADHD), and behavioral disorders (3). A previous study reported a substantial increase in the number of children diagnosed with mental disorders in the US before the onset of the COVID-19 pandemic (4), and the pandemic exacerbated the mental health conditions experienced by children due to factors such as social isolation, disruptions of routines and support systems, and limited access to mental health services (5).

Early childhood is a pivotal stage of child development (6). During this period, a child’s brain undergoes rapid growth and development (7). Positive experiences in early childhood contribute to emotional resilience, self-esteem, and development of crucial coping skills, whereas negative experiences can impair learning ability and relationships with others, causing lifelong complications (8). Because a child’s brain is highly adaptable and responsive to environmental influences during early childhood, it is imperative for young children with mental disorders to receive timely treatment, which can prevent more severe mental health problems or other chronic diseases in later life (9).

Many children with a mental disorder are untreated. The unmet needs for treatment among children with mental disorders likely reflect the complex interplay of individual, family, community, and societal factors (10), but the multilevel factors associated with not receiving needed mental health services among young children have not been identified. Addressing this gap in knowledge is essential to guide priority areas for strategies designed to improve the mental well-being of children in the US. The objective of this study was to assess the prevalence of early childhood mental disorders among US children after the COVID-19 pandemic and the proportion of these children not receiving mental health services when needed. An additional objective was to identify factors at the child, parental, household, neighborhood, and health care levels associated with not receiving needed mental health services among children with mental disorders in early childhood.

## Methods

We used nationally representative data for US children from the National Survey of Children’s Health (NSCH). The NSCH is a series of national surveys conducted by the US Census Bureau to assess the health and well-being of US children aged 0 to 17 years (11); the survey collects a wide range of data related to children’s mental health through online or mailed surveys. A parent or caregiver (referred to as “parent” hereinafter) familiar with the child’s health status and health care is the survey respondent. The 2 most recent cycles of NSCH (2021 and 2022) consisted of 104,995 children representative of noninstitutionalized children aged 0 to 17 years. The weighted percentage of children in age groups 0 to less than 2 years (infant to toddler), 2 to 8 years (early childhood), more than 8 to less than 12 years (middle childhood), and 12 to less than 18 years (adolescence) was 9.9%, 37.8%, 17.1%, and 35.2%, respectively. For this study, we included 46,424 children who were in early childhood at the time of the survey.

### Mental disorders

Parents were asked whether they had ever been told by a doctor or other health care provider that their child had any of 22 health conditions. A child was considered to ever have had a mental disorder if their parent responded yes to 1 or more of the following 10 conditions: depression, anxiety, behavioral and conduct problems, ADHD, autism spectrum disorder, Tourette syndrome, learning disability, intellectual disability, development delay, or language disorder. Parents who responded yes to any of these conditions were further asked whether this child currently had the condition.

### Not receiving health care or mental health services when needed

All parents were asked whether at any time in the previous 12 months their child needed health care but did not receive it. The child whose parent responded yes to this question was categorized as not receiving any needed health care in the previous 12 months, and these parents were further asked which type of health care (medical, dental, vision, hearing, mental health services, or others) was not received. The child whose parent marked “mental health services” was categorized as not receiving needed mental health services in the previous 12 months. In addition, these parents were asked whether any of the following 6 reasons contributed to this child not receiving needed health services: child not eligible for the services, services needed not available in the area, problems getting appointments, problems getting transportation or childcare, doctor’s office was not open, and issues related to cost.

### Child, parental, household, neighborhood, and health care factors

The NSCH Screener Questionnaire and Topical Questionnaire asked parents about various demographic, parental, household, neighborhood, and health care factors. For the children, we included the following variables: the child’s age, sex, race, ethnicity, nativity (born in US or outside US), and general health. For parental characteristics, we included parent’s age, education, nativity (first-, second, third-generation household or more, and other), and place of birth (born in US or outside US). For household characteristics, we included family structure, primary household language, and whether the household received cash assistance from government, food stamps or Supplemental Nutrition Assistance Program (SNAP) benefits, free or reduced-cost meals, or WIC (Special Supplemental Nutrition Program for Women, Infants, and Children) program benefits in the previous 12 months.

Neighborhood characteristics included the physical environment of the neighborhoods, such as whether there were sidewalks or walking paths, a park or playground, a recreation or community center, a library or book mobile, litter or garbage on street, poorly kept or rundown housing, and vandalism such as broken windows or graffiti in the neighborhood. Parents were also asked to what extent they agreed with the statement about the neighborhood offering a supportive or safe environment, such as people in the neighborhood helped each other out, watched out for each other’s children in the neighborhood, felt this child is safe in the neighborhood, or knew where to go for help in the community when encountering difficulties.

For children who had a health care visit in the previous 12 months, parents were asked how often the child’s doctors or other health care providers 1) spent enough time with this child, 2) listened carefully to parents, 3) showed sensitivity to the family’s values and customs, 4) provided the specific information parents needed concerning this child, and 5) helped parents feel like a partner in this child’s care. Response options were always, usually, sometimes, or never. For children who needed decisions to be made about their health care, such as whether to get prescriptions, referrals, or procedures, parents were asked how often the child’s health care providers 1) discussed with them the range of options to consider for this child’s health care or treatment, 2) made it easy for parents to raise concerns or disagree with recommendations for this child’s health care, and 3) worked with parents to decide which healthcare and treatment choices would be best for this child. Response options were always, usually, sometimes, or never.

For health insurance coverage, parents were asked whether their child was currently (in the previous 12 months) covered by health insurance. Parents who indicated having current insurance coverage were further asked about the type of insurance, and how often the child’s health insurance benefits met the child’s overall health needs and mental or behavioral health needs. For the 2 latter questions response options were always, usually, sometimes, or never. In addition, parents were asked whether the family had problems paying the child’s health care bills in the previous 12 months.

### Statistical analysis

We first examined the characteristics of US children in early childhood. We then estimated the weighted prevalence and 95% CIs of having 1 or more mental disorders among these children. We further estimated the proportion and associated 95% CIs of not receiving health care when needed, the type of health care services not received, and reasons for not receiving any needed health services, among all US children aged 2 to 8 years and separately for those with a current mental disorder. Among children with a current mental disorder, we compared the child, parental, household, neighborhood, and health care factors between those who did not receive needed mental health services and those who did; we used analysis of variance or χ^2^ tests for these comparisons. We used the Bonferroni correction to adjust for multiple comparisons (12).

We used survey weights to account for the complex sampling design of NSCH and nonresponse biases in generating nationally representative estimates. A 2-sided *P* value of ≤.05 denoted significance. We used SAS version 9.4 (SAS Institute, Inc) to conduct all analyses.

## Results

Among US children aged 2 to 8 years whose parents participated in the NSCH, the mean age was 5.0 years (Table 1). Of these children, 51.2% were boys, 12.7% were non-Hispanic Black, 25.5% were Hispanic, and 49.5% were non-Hispanic White; 96.5% were born in the US. Among those born outside the US, the mean length of time living in the US was 3.1 years. More than two-thirds (69.7%) of parents reported excellent general health for their child. Almost three-quarters (73.8%) of children were from households with 2 parents, and 84.5% of households spoke English as the primary language. Approximately one-fifth (21.0%) of households had received SNAP benefits and 14.0% had received WIC benefits in the previous 12 months.

**Table 1:** Child, Household, and Neighborhood Characteristics of Children Aged 2–8 Years in the United States (N = 46,424) Whose Parents Participated in the National Survey of Children’s Health, 2021–2022

Characteristic	% (95% CI)^a^
**Child**	
**Age, mean (95% CI), y**	5.0 (4.97–5.06)
**Sex**	
Male	51.2 (50.2–52.0)
Female	48.8 (47.8–49.8)
**Race and ethnicity**	
Hispanic	25.5 (24.5–26.6)
Non-Hispanic Black	12.7 (12.0–13.5)
Non-Hispanic White	49.5 (48.5–50.5)
Other^b^	12.2 (11.7–12.8)
**Child place of birth**	
In US	96.5 (95.9–97.0)
Outside US	3.5 (3.0–4.1)
**Length of time in the US among children born outside US, mean (95% CI), y**	3.1 (2.8–3.3)
**General health**	
Excellent	69.7 (68.7–70.6)
Very good	22.8 (22.0–23.7)
Good	6.2 (5.7–6.8)
Fair	1.2 (0.8–1.5)
Poor	0.05 (0.03–0.12)
**Parent**	
**Age, mean (95% CI ), y**	38.6 (38.4–38.8)
**Education**	
Less than high school	10.3 (9.5–11.2)
High school or GED	19.0 (18.1–20.0)
Some college or technical school	25.8 (24.8–26.7)
College degree or higher	44.8 (43.8–45.9)
**Place of birth**	
In US	76.1 (75.0–77.2)
Outside US	23.9 (22.8–25.0)
**Nativity^c^**	
First-generation household	2.6 (2.1–3.1)
Second-generation household	23.9 (22.9–24.8)
Third-generation household or more	66.6 (65.6–67.6)
Other	6.9 (6.3–7.5)
**Household**	
**Family structure**	
Two parents	73.8 (72.8–74.8)
Single parent	21.8 (20.9–22.8)
Grandparent household	3.2 (2.9–3.6)
Other	1.1 (0.9–1.3)
**Primary household language**	
English	84.5 (83.5–85.4)
Spanish	10.0 (9.1–10.9)
Other	5.6 (5.1–6.1)
**In the past 12 months, family has ever received:**	
Cash assistance from government	5.2 (4.7–5.7)
Food stamps or SNAP benefits	21.0 (20.0–21.9)
Free or reduced-cost meals	37.1 (36.0–38.1)
WIC benefits	14.0 (13.2–14.9)

Abbreviations: GED, General Educational Development; SNAP, Supplemental Nutrition Assistance Program; WIC, Special Supplemental Nutrition Program for Women, Infants, and Children.

a All values are percentage (95% CI) unless otherwise indicated; percentages and 95% CIs were adjusted for survey weights.

b Participants who were not Hispanic, non-Hispanic Black, or non-Hispanic White were grouped into the “Other” group.

c First-generation households are households where ≥1 parent was born outside the US and child was born outside the US; second-generation households are households where ≥1 parent is born outside the US and the child is born in the US; third-generation households or more are households where both parents were born in US; other households are households where child is born in the US and parents are not listed.

The prevalence of having ever received a diagnosis of an early childhood mental disorder was 19.0% (weighted n = 5,239,089), and the prevalence of currently having an early childhood mental disorder was 16.0% (weighted n = 4,405,414). The prevalence of having ever been diagnosed with a mental disorder was 11.2% for language disorder, 7.7% for development delay, 6.3% for behavioral and conduct disorder, 4.9% for learning disability, 4.6% for ADHD, 4.0% for anxiety, 3.3% for autism spectrum disorder, 0.8% for intellectual disability, 0.5% for depression, and 0.1% for Tourette syndrome (Table 2). The prevalence of currently having a mental disorder was 9.0% for language disorder, 6.2% for development delay, 5.4% for behavioral and conduct disorder, 4.4% for learning disability, 4.3% for ADHD, 3.4% for anxiety, 3.1% for autism spectrum disorder, 0.8% for intellectual disability, 0.5% for depression, and 0.1% for Tourette syndrome.

**Table 2:** Prevalence of Mental Disorders Among Children in Early Childhood (Aged 2–8 Years) in the US, National Survey of Children’s Health, 2021–2022

Disorder	N (weighted n)^a^	Weighted % (95% CI)^b^
**At least 1 mental disorder**		
Ever	8,714 (5,239,089)	19.0 (18.2–19.8)
Current	7,047 (4,405,414)	16.0 (15.2–16.7)
**Language disorder**		
Ever	4,975 (3,074,499)	11.2 (10.5–11.8)
Current	4,029 (2,486,755)	9.0 (8.4–9.6)
**Developmental delay**		
Ever	3,686 (2,116,305)	7.7 (7.2–8.2)
Current	2,912 (1,697,624)	6.2 (5.7–6.6)
**Behavioral or conduct problems**		
Ever	2,927 (1,743,197)	6.3 (5.9–6.8)
Current	2,582 (1,497,935)	5.4 (5.0–5.9)
**Learning disability**		
Ever	1,859 (1,344,023)	4.9 (4.4–5.4)
Current	1,776 (1,212,369)	4.4 (4.0–4.8)
**Attention deficit/hyperactivity disorder**		
Ever	1,942 (1,246,007)	4.6 (4.2–5.0)
Current	1,846 (1,193,736)	4.3 (4.0–4.7)
**Anxiety**		
Ever	1,933 (1,090,934)	4.0 (3.6–4.3)
Current	1,733 (950,394)	3.4 (3.1–3.8)
**Autism spectrum disorder**		
Ever	1,396 (899,389)	3.3 (2.9–3.6)
Current	1,334 (849,498)	3.1 (2.7–3.4)
**Intellectual disability**		
Ever	345 (218,759)	0.8 (0.6–1.0)
Current	329 (213,157)	0.8 (0.6–0.9)
**Depression**		
Ever	236 (169,285)	0.6 (0.5–0.8)
Current	197 (127,181)	0.5 (0.4–0.6)
**Tourette syndrome**		
Ever	58 (35,389)	0.13 (0.07–0.19)
Current	50 (26,672)	0.10 (0.05–0.15)

a N is the number of children whose parents participated in the National Survey of Children’s Health; weighted n is the number of US children represented by parents.

b Percentages and 95% CIs were adjusted for survey weights.

Among US children in early childhood, 3.0% (weighted n = 814,794) reported not receiving any needed health care in the previous 12 months (Table 3); of these children, 29.0% (weighted n = 230,315) reported not receiving mental health services when needed. Among children with a current mental disorder, 9.1% (weighted n = 400,095) reported not receiving any needed health care in the previous 12 months; of these children 45.8% (weighted n = 178,119) reported not receiving mental health services when needed. Overall, the prevalence of not receiving mental health services when needed was 0.8% among all US children in early childhood, and 4.0% among US children with a current early childhood mental disorder. The top reasons for not receiving any needed health care among children with a current early childhood mental disorder were problems getting appointment (72.1%), issues related to cost (39.3%), and needed services not being available in the area (38.5%).

**Table 3:** Prevalence of and Reasons for Children Aged 2–8 Years Not Receiving Health Services When Needed in the United States, National Survey of Children’s Health, 2021–2022^a^

Category	% (95% CI)	
	All children aged 2–8 years (n = 46,424)	Children aged 2–8 years with current mental disorders (n = 7,407)
**Not receiving any health care services when needed**	3.0 (2.6–3.3)	9.1 (7.8–10.5)
**Type of needed health services not received**		
Medical care	33.9 (28.0–39.9)	27.1 (18.9–35.3)
Dental care	45.7 (39.8–51.5)	33.7 (26.2–41.2)
Vision care	10.2 (7.1–13.4)	9.1 (4.9–13.3)
Hearing care	6.5 (3.7–9.3)	4.4 (2.0–6.8)
Mental health services	29.0 (24.3–33.6)	45.8 (38.2–53.5)
Other	13.5 (10.1–16.9)	18.5 (13.2–23.9)
**Not receiving mental health services when needed**	0.8 (0.7–1.0)	4.0 (3.3–4.8)
**Reasons for not receiving any needed health services**		
Problems getting appointment	60.1 (54.1–66.1)	72.1 (65.1–79.0)
Issues related to cost	37.6 (31.7–43.4)	39.3 (31.4–47.2)
Services needed not available in the area	29.4 (24.5–34.2)	38.5 (31.1–45.9)
Doctor’s office wasn’t open	21.1 (16.2–26.0)	21.8 (13.8–29.8)
Child not eligible for the services	18.8 (14.2–23.4)	18.5 (12.8–24.3)
Problems getting transportation or childcare	13.5 (9.9–17.1)	16.9 (11.0–22.8)

a Percentages and 95% CIs were adjusted for survey weights.

Among US children in early childhood with a current mental disorder, those who received and did not receive the mental health services when needed did not differ by any child, household, or neighborhood characteristic (Table 4). However, the parents of children who did not receive mental health services when needed, compared with the parents of children who received services, were significantly more likely to report worse experiences with health care providers in all 5 domains. For example, the percentage of parents who indicated that health care providers never spend enough time with their child was 11.5% among parents of children who did not receive needed mental health services and 2.1% among parents of children who received services (Table 4). In addition, parents of children who did not receive needed mental health services, compared with parents of children who received services, were significantly more likely to indicate that their child’s doctor or other health care provider never discussed the range of options for treatment (6.1% vs 2.9%), never made it easy to raise concerns or disagreements (5.1% vs 3.1%), or never worked with caregivers to decide together best treatment choices (12.7% vs 1.6%).

**Table 4:** Child, Household, Neighborhood, and Health Care Factors Associated With Not Receiving Mental Health Services When Needed Among US. Children with Mental Disorders in Early Childhood, National Survey of Children’s Health, 2021–2022

Characteristics	Receipt of mental health services when needed, % (95% CI)^a^		*P* value^b^
	Yes (n = 7,077)	No (n = 330)	
**Child**			
**Age, mean (95% CI), y**	5.9 (5.6–6.2)	5.5 (5.4–5.6)	.39
**Sex**			
Male	64.5 (62.2–66.8)	68.7 (60.2–77.3)	>.99
Female	35.5 (33.2–37.8)	31.3 (22.7–39.8)	
**Race and ethnicity**			
Hispanic	24.5 (22.1–27.0)	22.5 (14.9–30.1)	>.99
Non-Hispanic Black	15.1 (13.1–17.2)	14.0 (5.1–22.9)	
Non-Hispanic White	50.2 (47.8–52.6)	52.0 (42.7–61.4)	
Other	10.1 (8.8–11.4)	11.5 (6.1–16.8)	
**Place of birth**			
In US	97.5 (96.6–98.4)	98.7 (97.2–100)	>.99
Outside US	2.5 (1.6–3.4)	1.3 (0–2.8)	
**Household**			
**Hard to cover basic needs such as food or housing**			
Never	47.3 (44.9–49.7)	43.5 (34.3–52.7)	>.99
Rarely	32.7 (30.4–45.0)	30.4 (21.5–39.3)	
Somewhat often	15.7 (13.8–17.5)	19.0 (11.0–27.0)	
Very often	4.3 (3.4–5.2)	7.0 (3.3–10.8)	
**In the past 12 months, family has ever received**			
Cash assistance from government	6.7 (5.3–8.0)	4.9 (1.9–7.9)	>.99
Food stamps or Supplemental Nutrition Assistance Program benefits	29.5 (27.1–32.0)	28.0 (18.7–37.3)	>.99
Free or reduced-cost meals	48.4 (45.9–50.9)	48.9 (39.5–58.3)	>.99
Women, Infant, and Children (WIC) benefits	15.2 (13.3–17.1)	12.6 (6.5–18.8)	>.99
**Neighborhood**			
**Physical environment**			
Sidewalks/walking paths	73.7 (71.6–75.8)	71.1 (63.3–78.9)	>.99
Park/playground	73.9 (71.6–76.1)	74.6 (66.9–82.4)	>.99
Recreation center	45.8 (43.3–48.3)	43.1 (33.7–52.5)	>.99
Library/bookmobile	64.9 (62.5–67.3)	61.6 (52.4–70.7)	>.99
Litter/garbage	23.8 (21.6–26.0)	28.0 (19.3–36.7)	>.99
Rundown housing	16.7 (14.7–18.8)	18.1 (10.6–25.6)	>.99
Vandalism	9.3 (7.6–11.0)	9.2 (4.4–13.9)	>.99
**Supportive/safe environment**			
Help each other out	80.0 (77.5–81.9)	78.1 (69.6–86.6)	>.99
Watch out for other’s children	79.3 (77.2–81.3)	74.2 (65.3–83.1)	>.99
Know where to go for help	86.0 (84.2–87.8)	74.6 (65.6–83.5)	>.99
Child safe in neighborhood	91.5 (90.1–93.0)	87.0 (79.7–94.3)	>.99
**Health care factors**			
**Experience with health care providers**			
**Spending enough time**			
Always	55.0 (52.4–57.5)	38.2 (28.6–47.8)	.004
Usually	31.6 (29.1–34.0)	32.7 (24.3–41.1)	
Sometimes	11.3 (9.7–13.0)	17.6 (10.7–24.5)	
Never	2.1 (1.5–2.7)	11.5 (5.2–17.9)	
**Listen carefully**			
Always	64.9 (62.4–67.4)	45.9 (36.3–55.4)	.004
Usually	27.3 (24.9–29.7)	29.6 (21.4–37.8)	
Sometimes	6.8 (5.5–8.1)	19.6 (12.2–27.0)	
Never	1.0 (0.6–1.6)	4.9 (0.6–9.2)	
**Show sensitivity to family values/customs**			
Always	68.0 (65.5–70.5)	56.5 (47.1–65.8)	.004
Usually	24.1 (21.8–26.4)	23.8 (16.1–31.4)	
Sometimes	6.4 (5.2–7.6)	14.7 (8.2–21.1)	
Never	1.5 (0.8–2.1)	5.1 (0.7–9.5)	
**Provide specific information needed**			
Always	65.0 (62.5–67.5)	38.7 (29.3–48.2)	.008
Usually	27.0 (24.6–29.4)	34.5 (25.7–43.3)	
Sometimes	6.9 (5.5–8.2)	21.8 (14.1–29.5)	
Never	1.2 (0.7–1.6)	5.0 (0.9–9.1)	
**Helped parents feel like partners in child’s care**			
Always	66.2 (63.7–68.7)	45.4 (35.8–54.9)	.004
Usually	24.4 (22.1–26.8)	30.4 (21.8–39.0)	
Sometimes	7.5 (6.1–9.0)	14.9 (8.7–21.1)	
Never	1.8 (1.1–2.6)	9.3 (3.9–14.7)	
**Discuss range of options**			
Always	59.7 (55.8–63.7)	37.7 (27.3–48.1)	.004
Usually	26.8 (22.9–30.7)	25.0 (16.4–33.6)	
Sometimes	10.6 (8.3–12.9)	31.2 (20.6–41.8)	
Never	2.9 (1.7–4.0)	6.1 (1.1–11.1)	
**Make easy to raise concerns**			
Always	62.3 (58.5–66.2)	42.0 (31.2–52.8)	.004
Usually	25.3 (21.7–29.0)	24.2 (15.8–32.6)	
Sometimes	9.3 (7.5–11.0)	28.7 (18.6–38.9)	
Never	3.1 (1.8–4.3)	5.1 (0.7–9.4)	
**Work together to decide best treatment**			
Always	64.1 (60.2–68.0)	42.1 (31.5–52.6)	.004
Usually	24.9 (21.2–28.6)	23.6 (14.9–32.4)	
Sometimes	9.4 (7.3–11.5)	21.6 (12.8–30.3)	
Never	1.6 (1.0–2.2)	12.7 (4.7–20.8)	
**Health insurance and payment**			
**Current covered by health insurance**			
Yes	96.6 (95.6–97.7)	97.6 (95.1–100)	>.99
No	3.4 (2.3–4.4)	2.4 (0–4.9)	
**Current insurance type**			
Through employer	53.0 (50.5–55.5)	57.6 (28.0–67.2)	>.99
Purchased directly from insurance company	4.2 (3.4–5.0)	4.0 (1.4–6.7)	>.99
Medicaid/Medicaid Assistance/government assistance plan	49.5 (47.1–52.0)	50.2 (40.7–59.7)	>.99
Indian Health Service	0.8 (0.4–1.2)	0.3 (0–0.5)	>.99
TRICARE/other military	2.8 (2.1–3.5)	3.9 (1.4–6.3)	>.99
Other	2.2 (1.4–2.9)	2.1 (0.4–3.7)	>.99
**Health insurance offers benefits or cover services that meet needs**			
Always	58.8 (56.4–61.2)	33.9 (24.5–43.2)	<.004
Usually	31.2 (29.0–33.5)	38.7 (29.8–47.6)	
Sometimes	8.6 (7.3–10.0)	21.5 (14.4–28.7)	
Never	1.3 (0.9–1.8)	5.9 (0–11.8)	
**Health insurance offers benefits or cover services that meet the mental or behavioral health needs**			
Always	53.1 (50.2–56.0)	17.5 (9.5–25.4)	<.004
Usually	28.2 (25.6–30.7)	24.7 (16.2–47.3)	
Sometimes	14.2 (12.1–16.2)	38.0 (28.7–47.3)	
Never	4.6 (3.2–5.9)	19.8 (12.1–27.6)	
**Problems paying health care bills in the past 12 months**			
Yes	21.3 (18.7–23.8)	32.0 (22.8–41.3)	.55
No	78.7 (76.2–81.3)	68.0 (58.7–77.2)	

a Percentages and 95% CIs were adjusted for survey weights.

b *P* values were generated from analysis of variance for continuous variables (age) and χ^2^ test for categorical variables (all others) and were adjusted for multiple comparisons using Bonferroni correction.

Children with an early childhood mental disorder who received needed mental health services did not differ from children who did not receive services by current health insurance coverage or type of insurance coverage. However, parents of children who did not receive needed mental health services, compared with parents of children who received services, were significantly more likely to indicate that their health insurance never offered benefits or covered services that meet needs (5.9% vs 1.3%) and that their health insurance never offered benefits or covered services that meet their mental or behavioral health needs (19.8% vs 4.6%).

## Discussion

In this nationally representative sample of US children, we found that 19.0% of US children in early childhood had ever received a diagnosis of a mental disorder and 16.0% had a current mental disorder. Among children with current early childhood mental disorders, 9.1% did not receive any needed health services and 4.0% did not receive needed mental health services in the previous 12 months. The lack of access to health care services, negative experiences with health care provides, and health insurance not covering the needed services were the factors most strongly associated with not receiving needed mental health services among these children.

The prevalence of ever having a mental health disorder among children aged 2 to 8 years in our study (19.0%) is a 9% relative increase and a 1.6 percentage-point absolute increase in the prevalence of mental disorders in children of this age since 2016, which was 17.4% (13). Mental disorders in early childhood can affect a child’s development and well-being, potentially resulting in long-term cognitive, emotional, and social challenges (14–18). The high and increasing prevalence of mental disorders among young children underscores the urgency of providing timely treatment during the critical period of child development. Among children aged 2 to 8 years, the difference in the prevalence of receiving any health care services when needed between children with current mental disorders in early childhood and all children (9.1% vs 3.0%) was largely driven by a high proportion of children with mental disorders who reported that they did not receive the needed mental health services in the previous 12 months. Despite the rapid advancements in evidence-based treatment of children with mental disorders (19,20), children with early childhood mental disorders may encounter more barriers to receiving health care when needed, particularly mental health services, compared with children without mental disorders.

The top reasons for not receiving any needed health care services among children with early childhood mental disorders were similar to reasons among all children aged 2 to 8 years. However, the percentage of children with mental disorders in early childhood who had problems related to appointment availability and service accessibility was higher than the percentage of all children aged 2 to 8 years, suggesting that poor access to health care services may be a systemic barrier. Our findings indicated that 72.1% of children with mental disorders who did not receive any needed health care reported difficulties in getting appointments, and nearly 40% did not have needed services available in their areas. Future strategies need to focus on bridging the gap between the demand and availability in mental health services for young children with mental disorders.

In our effort to identify factors associated with unmet needs, we assessed a wide range of factors, following the social-ecological model for health (21). Despite previously reported disparities by child and family socioeconomic position and household (22) or neighborhood conditions (23), we did not find significant differences in receiving needed mental health services by child, household, or neighborhood factors. Instead, we found that poor experiences with health care providers were strongly associated with not receiving needed mental health services. In particular, parents of children who received needed mental health services, compared with parents of children who did not, were 5 times more likely to report that their health care providers never spent enough time, never listened carefully, or never helped them feel like a partner in their child’s health care. Our findings align with previous research that assessed barriers to seeking and receiving mental health services, including the feeling of not being listened to or being dismissed by health care professionals (24). Together, these findings suggest that trust and confidence in health service providers could play a crucial role in parents seeking mental health services for children in need (25).

Lack of insurance coverage has been reported as a barrier to accessing mental health services in previous studies (26). However, we found that young children with mental disorders who received health care services and young children with mental disorders who did not receive health care services had similar rates of health insurance coverage, regardless of insurance type. However, children who did not receive needed mental health services were more likely than children who did to report that their insurance did not allow them to see the health care providers they needed or offer mental health benefits. Thus, despite the overall similar rates in insurance coverage, certain insurance plans may not provide adequate benefits to meet the child’s mental health service needs. The cost of mental health services may also be a barrier to receiving them. Although we did not find significant differences between those who received or did not receive needed mental health services, 20% to 30% of parents of children with mental disorders in early childhood reported having difficulties paying the child’s health care bills.

### Strengths

Our study has several strengths. It provides updated evidence on the prevalence of mental disorders among US children after the COVID-19 pandemic. The analyses used data from a nationally representative sample of US children. Our findings, therefore, are generalizable to all noninstitutionalized children in the US. The focus on children in early childhood has important implications, because many mental disorders start in early childhood, and early interventions can substantially reduce the risk of future long-term complications. In addition, our study analyzed data from NSCH, one of the few national surveys that provide comprehensive surveillance data on a wide range of mental disorders among young children, such as those in early childhood.

### Limitations

Our study has some limitations. First, the cross-sectional design of this study prevents us from establishing causal relationships. Second, the assessment of mental disorders was based on parental self-report, which is subject to recall bias or social desirability bias. In addition, the parent’s self-reported need for mental health services may not reflect the need assessed by health care providers. Third, the NSCH lacks adequate representation of children from racial and ethnic groups other than non-Hispanic White, non-Hispanic Black, and Hispanic groups, and does not include a sufficient number of children who speak languages other than English or Spanish. These factors limit our ability to assess whether language or culture acts as a barrier to seeking and receiving mental health services among children with mental disorders.

### Conclusion

Our study provides updated evidence on the prevalence of early childhood mental disorders among US children after the COVID-19 pandemic. With the high and increasing prevalence of mental disorders among young children, it is imperative to improve the provision of mental health services for those in need. In addition to increasing the availability of mental health services and expanding health insurance coverage, future public health efforts should prioritize enhancing patients’ experiences with health care providers and establishing patient-centered communications on children’s mental health needs and treatment options.
